# Supplementary material for: Cross-cultural adaptation and psychometric evaluation of the urdu version of the morisky, greene, and levine medication adherence scale (MGLS-4) for major depressive disorder patients
Source: PLoS One. 2025 Apr 28;20(4):e0320258. doi: 10.1371/journal.pone.0320258 (PMC12036920; doi:10.1371/journal.pone.0320258)
Supplement: S1 Fig — (PDF) [file pone.0320258.s001.pdf]

Supporting information

Table S1: Urdu version of MGLS-4

UMGLS-4	MGLS-4 Original (Morisky et al., 1986)	
کیا آپ کبھی اپنی ادویات استعمال کرنا بھول جاتے ہیں؟	Do you ever forget to take your medicine?	**Q1**
کیا آپ کبھی ادویات کے استعمال میں بے احتیاطی برتتے ہیں؟	Are you careless at times about taking your medicine?	**Q2**
کیا آپ اپنی مرض میں بہتری محسوس کرنے پر ادویات کا استعمال کبھی کبھار ترک کر دیتے ہیں؟	When you feel better do you sometime stop taking your medicine?	**Q3**
کیا کبھی مرض کی شدت میں اضافے کی صورت میں آپ اپنی ادویات کا استعمال بند کر دیتے ہیں؟	Sometimes if you feel worse when you take the medicine, do you stop taking it?	**Q4**
