# Supplementary material for: Cross-cultural adaptation and psychometric evaluation of the urdu version of the morisky, greene, and levine medication adherence scale (MGLS-4) for major depressive disorder patients
Source: PLoS One. 2025 Apr 28;20(4):e0320258. doi: 10.1371/journal.pone.0320258 (PMC12036920; doi:10.1371/journal.pone.0320258)
Supplement: Attachment — Submitted filename: Response to reviewers comments.docx [file pone.0320258.s004.docx]

# Subject: Response to Review Comments for Manuscript ID: PONE-D-24-26034

**Dear Marco Innamorati,**

Thank you for the opportunity to revise our manuscript, "*Cross-Cultural Adaptation and Psychometric Evaluation of the Urdu Version of the Morisky, Greene, and Levine Medication Adherence Scale (MGLS-4) for Major Depressive Disorder Patients*," and for the insightful comments from the reviewers. We have carefully considered each comment and have made corresponding changes to our manuscript. Below, we provide a detailed response to each comment:

**Reviewer #1:**

| **Section** | **Reviewer Comments** | **Author’s Response** |
| --- | --- | --- |
| **Introduction** | The introduction is well-structured, giving a clear overview of the problem. However, it would be beneficial if there were closer links between the need for the cultural and linguistic validation of UMGLS-4 and the clinical challenges observed in Pakistan. Emphasizing, for instance, the potential practical implications of the validated scale could make the introduction more appealing. | Thank you for valuable suggestions. Enhanced introduction to connect UMGLS-4 validation with clinical challenges in Pakistan.  Page: 6, Line: 115-121 |
| **Objectives** | Objectives are clear and pertinent. However, elaborating on the clinical significance of validating the UMGLS-4 would amplify its relevance. For instance, discussing how this tool would be utilized in a clinical setup for advancing medication adherence strategies for MDD patients. | Expanded objectives to detail UMGLS-4's clinical application  Page 6, Line: 121-123 |
| **Methodology** | 1. Sampling: Convenience sampling is used, which is fine, but this should be discussed in a more elaborative manner, for instance, how would this limit the generalization of the findings in rural or socioeconomically diverse populations. | Discussed limitations and implications of convenience sampling on generalization.  Section: Discussion Page:25, Lines: 376-382 |
|  | 2. Translation Process: The elaborate translation and back-translation process is really appreciable. | Thank you |
|  | 3. Statistical Analyses: The application of non-parametric tests, such as Kruskal-Wallis and Mann-Whitney U, is appropriate but requires justification. Were assumptions of normality and homoscedasticity tested, for example, the Shapiro-Wilk test? This would enhance the appropriateness of the statistical method. | Provided Shapiro-Wilk test results to justify non-parametric methods.  Section: Statistical Analyses Page: 10, Line: 202-203 |
|  | 4. Factor Analysis: Although EFA and CFA were performed, the Kaiser-Meyer-Olkin (KMO) index of 0.662 indicates only moderate sampling adequacy. Limitations of the study need to be declared explicitly. | Included the limitation in the discussion section  Section: Discussion Page: 25, Lines: 384-385 |
| **Results** | 1. Medication Adherence Trend: The demographic variations, though explained, would still be easier to understand when summarized using visual aids (such as bar graphs or pie charts) that highlight their key differences. | Included a Bar Chart demonstrating medication adherence status using UMGLS-4 and DAI-10, as shown in Figure 1.  Page: 14 |
|  | The observed major association with education is highly intriguing but the result does not relate it to any hypothesis. What could explain why education will make a difference here? Also, influence from employment status on adherence must also be explored further. | Clarified in with deeper insights in discussion section  Page: 23, Lines: 350-357 |
|  | 2.Regression Analysis: The multivariate regression analysis is useful, but key metrics such as the coefficient of determination ( R^2 ) and the overall model significance test (F-test) are missing. Including these would provide a clearer picture of the model’s explanatory power. | Added key metrics such as R^2 and F-test to clarify the model's explanatory power.  Page: 22, Lines: 308-313 |
|  | While education emerges as a significant predictor, the lack of significance for other variables (e.g., gender, occupation) raises questions. Could collinearity or insufficient sample size have affected these results? Including the VIF would complete the concern of multicollinearity. | Included VIF in Regression analyses and as well as summarized in Table 6.  Page: 22 |
| **Discussion** | The discussion is well-expressed and puts the findings into the perspective of the existing literature. However, it has a heavy leaning towards comparing findings with those of previous studies. There is a need for deeper reflection on what the findings mean in terms of practice. For example, how might clinicians or policy makers take this tool and use it to enhance adherence in similar settings? | Enhanced discussion to reflect deeper on how findings can be practically applied by clinicians and policymakers to improve adherence.  Pages: 23-24, Lines: 366-375 |
| **Conclusions** | The conclusions are well-written, but could be stronger in driving home the usefulness of the UMGLS-4 by explicitly discussing its potential role in guiding interventions to improve | Revised conclusions to strongly emphasize the role of UMGLS-4.  Pages: 24-25 |

**Reviewer #2:**

| **Section** | **Comments** | **Response** |
| --- | --- | --- |
|  | Throughout the manuscript, there are several typographical errors: periods following commas (e.g., line 57; p.8), spaces before periods (e.g., line 110; p.12), extra spaces (e.g., lines 159, 179, 199; pp. 14, 15, 16 respectively), commas instead of periods (e.g., line 210; p.16), and missing spaces (e.g., line 214; p.16). | Corrected all typographical errors and standardized formatting across the manuscript, particularly for the presentation of p-values.  Throughout the manuscript |
|  | Furthermore, the writing style should be standardized. For example, p-values are italicized in some parts of the manuscript but not in others. It would be better to italicize all p-values, and it is not necessary to specify the term "value" in brackets, you could simply write (p=/...). | Thank you for valuable suggestions. Changed Throughout the manuscript |
|  | When presenting suggestions without references to support the statements, it wold be preferable to use conditional verbs (e.g., lines 97-98; p.10: “[…] it could be crucial”). | Thank you for your kind suggestion. Changed  Page: 5, line: 96 |
| **Abstract & introduction:** | - In the abstract, the term “behaviour” appears (line 41; p.8), while elsewhere in the manuscript the term “behavior(s)” is used. I recommend standardizing the text to a consistent linguistic form, and reviewing whether other terms also need standardization. | Changed throughout the Manuscript, and “behavior” is used. |
| **introduction** | , I suggest providing a reference and page number for the citation reported in lines 84-85 (p. 10). | Changed and updated the citation |
|  | Additionally, in line 86 (p. 11) the second occurrence of “Medication nonadherence” can be omitted, leaving only the acronym, as the term has already been introduced in full earlier. | Acronym Left  Page: 4, Line: 84 |
|  | It would also be helpful to clarify or rewrite line 89 (p. 11), when there is reported “This difference”, it does not make clear what difference is being referenced. | Changed and clarified  Page:4, Line: 87 |
| **Methodology** | In the “Study population and sampling” paragraph, I suggest explicitly stating that the inclusion and exclusion criteria are been reported (lines from 130 to 133; pp. 12-13). | Inclusion and Exclusion criteria are explicitly stated in “Study population and sampling”  Page 7, Lines: 132-135 |
|  | Furthermore, it would be preferable to provide more details regarding the sampling methods, such as who carried out the recruitment, how it was conducted (e.g., in-person or by telephone), when patients were recruited (e.g., during pre-scheduled visits or during their hospitalizations). | Addressed as per comments |
|  | In the “Translation and Cultural Adaptation of MGLS-4 into Urdu” paragraph, I recommend writing out the full name of the tool and including the acronym in parentheses (line 139; p. 13). | Changed according to comment  Page: 7, Lines: 140-141 |
|  | In the “Data collection and questionnaires” paragraph, specify how the data were collected (e.g., via online systems or paper forms - in the latter case, who supervised the process). | Added the details according to the comment  Page: 8  Line: 165-166 |
|  | Additionally, I suggest reporting the Cronbach’s alpha of your study for the DAI-10 to ensure reliability is clearly communicated. | Also mentioned Cronbach's Alpha Value as per the comment  Page: 8, Line: 168 |
|  | In the “Statistical analyses” paragraph, I suggest simplifying lines 179-180 (p.14) writing “demographic and clinical characteristics, and …”. | Simplified and clarified the line. |
| **Results** | In line 229 (p. 18) I suggest removing the period after “2” (Table 2.) and deleting the final sentence, as the table is already presented above. | The period was removed, and the last line was deleted as per the suggestion |
|  | In line 304 (p.26), I suggest including the zero before the decimal in B-value (i.e., B0.301). | Added 0 in B values  Line:  316 |
| **Discussion & conclusion** | these sections are well written. I suggest beginning the Discussion with one or two brief sentences summarizing the background of the study. | Revised the discussions and conclusions summarizing the background of study |

These revisions have addressed the concerns raised during the review process and strengthened the manuscript. We appreciate the reviewers' suggestions, which have undoubtedly improved the quality and clarity of our work.

We look forward to your decision and hope our revised manuscript will meet your expectations.

Thank you for considering our submission.

Sincerely,

**Dr. Fazli Khuda**

Assistant Professor

Department of Pharmacy

University of Peshawar, Pakistan

Email: [fazlikhuda@uop.edu.pk](mailto:fazlikhuda@uop.edu.pk)
